# Factors associated with sexual violence among waitresses working in Bahir Dar City, Ethiopia: a mixed-method study

**DOI:** 10.1186/s12905-022-01806-x

**Published:** 2022-06-06

**Authors:** Mulugeta Dile Worke, Habtamu Demelash, Lealem Meseret, Minale Bezie, Fantu Abebe

**Affiliations:** 1 Department of Midwifery, College of Health Sciences, Debre Tabor University, Debre Tabor, Ethiopia; 2 Department of Social and Public Health, Debre Tabor University, Debre Tabor, Ethiopia; 3 Department of Gynecology and Obstetrics, Debre Tabor University, Debre Tabor, Ethiopia; 4 Jhpiego-Ethiopia, Bahir Dar, Ethiopia

**Keywords:** Workplace sexual violence, Perception, Women, Ethiopia

## Abstract

**Background:**

When a worker is abused, threatened, or assaulted while at work, it poses an explicit or implicit threat to his/her safety, well-being, or health. However, the magnitude and understanding of the problem and its associated factors have received little attention in low and middle-income countries, including Ethiopia. Thus, this study aimed to ascertain the magnitude, perceptions, and associated factors of workplace sexual violence among waitresses in Bahir Dar, Ethiopia.

**Methods:**

Parallel sampling was used in a facility-based convergent mixed-methods study. A multistage sampling technique was used to select study participants. Four hundred six waitresses provided quantitative information. To collect data, a pretested structured interview administered questionnaire was used. Qualitative data from ten in-depth interviews and six focus group discussions were also collected using a semi-structured questionnaire. The data were cleaned before being entered into Epidata version 7 and exported to STATA version 15 for analysis. Using odds ratios with a 95% confidence interval and a *P* value of less than 0.05, binary logistic regression was used to identify independent predictors. Thematic analysis was performed using ATLAS ti version 8.4.25 after the recorded audios were transcribed.

**Results:**

The overall prevalence of workplace sexual violence was 45.9% (95% CI 41, 50.8). Waitresses who had heard about sexual violence and workplace sexual violence, had witnessed family violence as a child, had a family who valued their honor over their children’s safety and health, and lacked employment opportunities were more likely to experience workplace sexual violence. Waitresses who worked in emotionally supportive work environments and were educated were less likely to experience workplace sexual violence.

**Conclusions:**

Many of the waitresses in this study had experienced workplace sexual violence. Its awareness, witnessing family violence as a child, growing up in a family that prioritized their honor over their children’s safety and health, and lack of employment opportunities exacerbated it. In contrast, emotionally supportive work environments and education have protected them. This implies that organizations, both governmental and non-governmental, civic organizations, and other responsible bodies must pay attention to the identified factors. Additionally, Ethiopian hospitality workplaces should develop policies to protect women.

**Supplementary Information:**

The online version contains supplementary material available at 10.1186/s12905-022-01806-x.

## Introduction

Sexual violence (SV) is defined as a sexual assault committed or attempted by another person without the victim’s consent or against someone who is unable to consent or refuse [1]. It includes forced or alcohol/drug-assisted infiltration of a victim, non-physically coerced unwanted penetration, purposeful sexual touches, and non-contact sexual activities [2]. Every day, nearly 4,400 people die, and thousands are injured or suffer other non-fatal health consequences because of being the victim or witness of violent acts [3]. Tens of thousands of lives were lost, families were shattered, and enormous costs were incurred in treating victims, supporting families, restoring infrastructure, prosecuting perpetrators, or losing productivity and investment [3, 4].

Sexual violence can occur in any workplace and in both sexes. However, it is more common in women [4]. It is particularly common among women in certain job categories. According to studies, one out of every four female employees in the hospitality and tourism industries had been sexually violated [5, 6]. In Ethiopia, approximately 20% of the waitresses working in hotels and restaurants have experienced sexual violence [7]. According to an Ethiopian demographic and health survey, 11% of ever-married women have experienced sexual violence [8].

Furthermore, SV can have a variety of consequences, including psychological aggression across all employment sectors [1, 9, 10]. It is also a common precursor to mental illness and, to a lesser extent, physical injury among those who are exposed to this occupational hazard [11]. Additionally, it is a precursor to staff turnover, absenteeism, employee disengagement, a decrease in productivity and organizational commitment, and an increase in financial and economic costs, all of which lead to poor customer service. Furthermore, it may contribute to the industry’s poor reputation by creating a hazardous working environment [12, 13].

Many factors influence the likelihood of becoming a victim of sexual violence, including job category, the nature of the work being performed, gender, age, and experience [14]. It was also linked to workplace traits and working conditions [15]. These workplaces include male-dominated jobs such as corporate headquarters, semi-truck drivers, offices of healthcare providers, rural farms, and factories [13] traditionally. Waitresses’ perception is also important for their protection and aggression. Therefore, in high-risk environments for sexual violence, sexual and reproductive health management is critical for reducing physical, psychological, social, and reproductive health complications.

Sexual violence against waitresses is a persistent problem that has received insufficient attention. However, changes in the legal landscape regarding waitress sexual violence have increased the potential liability for employers, particularly those in the cafeteria, restaurants, food and beverage services, and other services that require the hiring of waitresses. But employers should not overlook the importance of developing effective anti-violence policies and improving organizational practices that limit a company’s legal exposure while protecting our country’s waitresses in the face of increased litigation and decreasing tolerance by the courts.

Therefore, this research attempted to provide information on its magnitude, risk factors, and health and consequences. This could help describe the extent of the problem for policymakers and donors. It would also help prepare the necessary resources and promote programs for better reproductive health care and address the information gap about factors associated with sexual violence among waitresses, allowing clients and stakeholders to intervene in such aspects. Furthermore, this study is expected to benefit a broad range of stakeholders.

## Methods and materials

### Study setting and period

This mixed-methods study was conducted among waitresses working in hotels, restaurants, beverage groceries, and cafeterias in Bahir Dar city between 1st January and 30th August 2019. Bahir Dar, the capital of Amhara National Regional State, is located 565 km northwest of Ethiopia’s capital. The city has a total population of 280,780 and is one of the country’s tourist destinations. The number of people in Bahir Dar is projected to increase by more than four times by 2040 [9]. The city is divided into 17 kebeles (a minor administrative structure in the country). Approximately 50 hotels at various levels, 62 restaurants, 41 beverage groceries, and over 88 medium and small cafeterias were estimated to be present in the city [16]. The number of waitresses working across these facilities ranged from four to twelve.

### Study design

This cross-sectional parallel mixed-methods study was conducted among women working in the hospitality industry. A phenomenological qualitative study was conducted with waitresses who had not participated in the quantitative study.

### Population

The study population included all randomly selected waitresses working in selected facilities using the census and those over 18 years of age. All waitresses working in hotels, restaurants, beverage groceries, and cafeterias in Bahir Dar were the source population. To reduce selection bias and excessive inflation of the magnitude of WSV, waitresses who had been working in nightclubs and commercial sex workers were excluded.

For the qualitative part, ten in-depth interviews (IDIs) and six focus group discussions (FGDs) were conducted with female hospitality workplace workers [17, 18]. These workers had at least six months of working experience in the hospitality industry, which was different from the quantitative study participants. The participants were working in hospitality workplaces in the study area. Women working in hospitality workplaces were identified and asked to be questioned. Community workers from non-governmental organizations living in the city where the study participants live assisted us in reaching the study participants. Ten female participants participated in the IDIs, and 35 female hospitality workplace workers participated in the FGDs. Five participants were in two FGDs, seven were in one, and six were in the other three (Additional file 1, Additional file 2).

### Sample size determination and sampling procedures

A sample size of 406 was determined using a single population proportion formula with the following assumptions: A study conducted in 2009 [7] found a prevalence (p) of 20% of waitresses sexually violated, a confidence interval (CI) of 95%, and a confidence limit (d) of 5% with a 10% non-response rate and a design effect of 1.5.

**Fig. 1:**
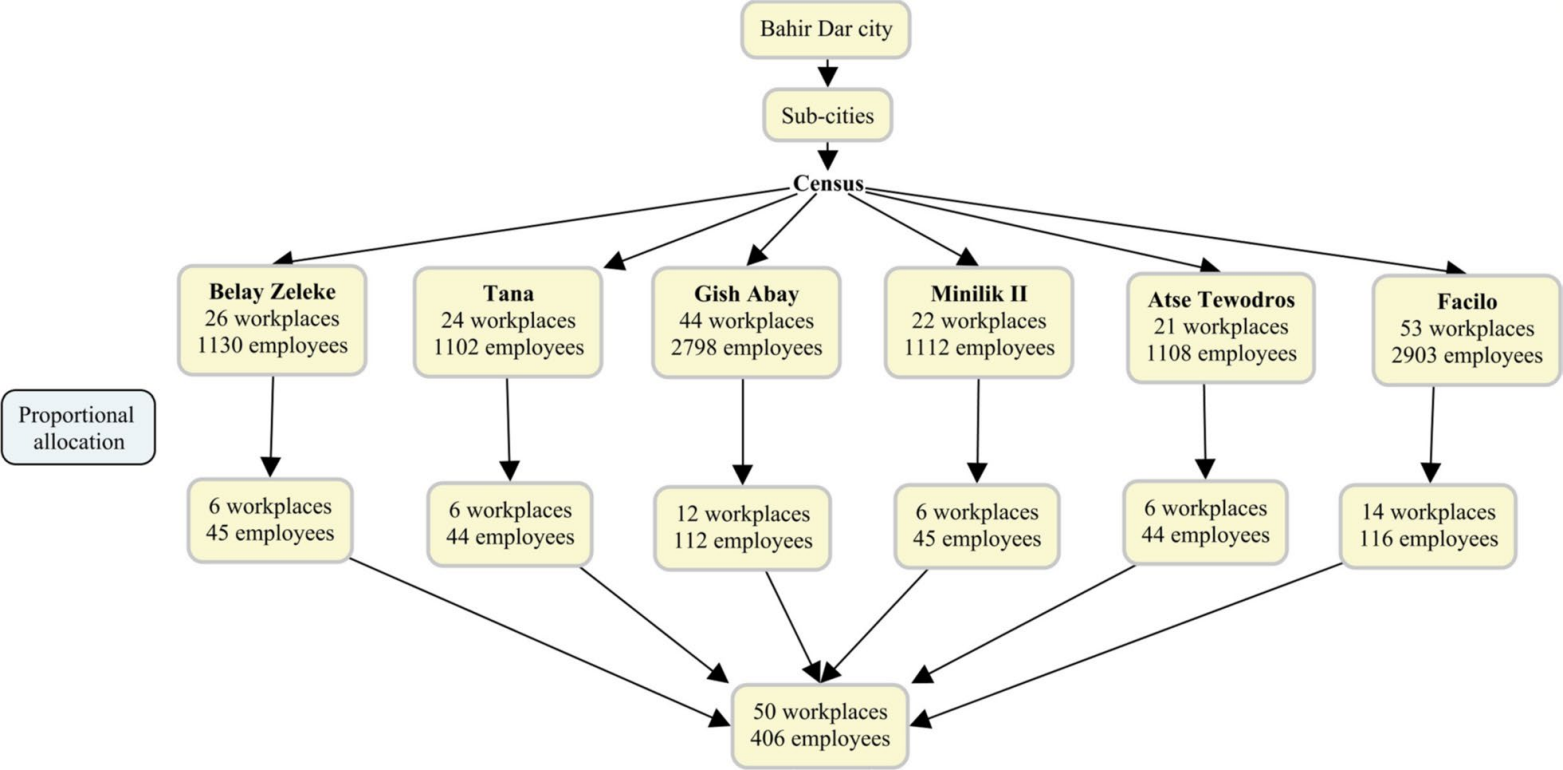
Sampling procedure for hospitality workplaces and women employees in Bahir Dar City Administration, Ethiopia, January to August 2019

A multistage sampling technique was used to obtain the study population. First, the city was stratified into six sub-cities based on administrative classification. A census was then conducted to get a list of hospitality workplaces and the number of female employees within each workplace in the six sub-cities. During the survey, workplaces that provided hospitality services to the community were identified. All eligible hospitality organizations deemed functional for at least six months before the survey were included. Different codes were provided to organizations and women. Fifty organizations were selected randomly, and female employees from each facility were determined based on a probability proportional to size using the pool of female employees registered during the census. Finally, employees were systematically selected using an organization-based census registry (Fig. 1).

After purposely identifying the first women for the qualitative data, women who worked in hospitality workplaces and experienced WSV were identified and contacted using the snowball method. Enrollment of the study participants was continued until the data were saturated. Non-governmental organizations’ community workers living in the city where the study participants live help us reach them.

### Variables and measurement

Sexual violence experiences, the companies they work for, their knowledge of any policies and procedures related to sexual violence, individual factors, relationship-related factors, community-related factors, and social-related factors were assessed. The dependent variable was sexual violence experience, and the independent variables were sociodemographic variables related to age, ethnicity, level of education, work experience, the department they were working in, and their position.

Risk factors for perpetration, including individual, relationship, community, and social factors, were measured based on the variables found through a detailed literature review [19–21]. Accordingly, six questions were used to assess sexual violence in this study. Over the previous month, participants were asked whether they had encountered (1) verbal abuse, (2) unwanted sexual attention, (3) threats and behavior meant to humiliate them, (4) physical assault, (5) bullying/harassment, or (6) sexual harassment over the previous 12 months. If they answered yes to either of these six questions, the respondents were deemed sexual violence victims.

Based on the recent study conducted by Casey and Masters [22], the awareness of sexual violence and workplace sexual violence, habits of alcohol drinking, history of family violence as a child, having sexually aggressive and delinquent peers, and growing up in a low-income family environment were measured [22]. Similarly, an emotionally supportive work environment, family consideration of honor over child safety and health, lack of employment opportunities, weak sanctions against sexual violence, and societal norms supportive of sexual violence were also measured. Similarly, the societal norm of supporting male superiority, weak laws and policies related to sexual violence, witnessing high crimes and other forms of violence in the community, and educational statuses were assessed. The response options for all the questions were 0 for no and 1 for yes. The length of the employment contract (one month, one month to one year, or more than one year), the occupation (managers, supervisors, cashiers, and waitresses), the number of hours worked each week, and other work-related parameters were also measured.

### Operational definitions

#### Sexual violence

Unwanted penetration through the use of force or the facilitation of alcohol/drug (e.g., rape), pressured sex (e.g., sexual coercion), unwanted sexual contact (e.g., groping), and non-contact unwanted experiences (e.g., unwanted sexual remarks) in specific settings, such as workplaces [23, 24]. Sexual violence was declared while they answered yes to one of the mentioned encounters.

#### Rape

any non-consensual penetration of the vagina, penetration obtained by physical body harm, threatening or deception, or when the victim cannot consent [25–28]. The response options were 0 = no, and 1 = yes.

#### Attempted rape

It is a trial to have sex by coercion, threatening, or deception, or when the victim cannot consent, but without actual penetration of the vagina [2, 4, 13, 25–28]. The response options were 0 = no, and 1 = yes.

#### Sexual harassment

Unwanted sexual behaviors, including jokes, verbal comments, and physical contact, are intentionally performed by the perpetrators on women or girls [17, 29]. The response options were 0 = no, and 1 = yes.

#### Witnessed inter-parental violence as a child

This is considered if the respondent had ever seen physical violence between her parents or adults who raised her before she was 14. The response options were 0 = no, and 1 = yes.

### Data collection technique and quality assurance

Ten female and two male public health professionals were recruited for the data collection and supervision. Recruitment was based on previous data collection (primarily on sensitive topics) and fluency in the local language. The enumerators (data collectors) and supervisors received three-day intensive training to conduct the enumeration, identify the women, collect the data, and supervise to assure data quality. The objectives and instruments of the study were presented by answering all questions. Following this review, questionnaire amendments and necessary modifications were made. The training also included obtaining written consent, conducting face-to-face interviews, and navigating the questionnaires. The three days of training were followed by one day of fieldwork and one day of final tool discussions.

The hiring process was mainly conducted through informal networks, and the target groups were not covered by the formal social security schemes of the local government. This results in the total number of waitresses working in an unknown area. Thus, a census was conducted for each of the five sub-cities. Two weeks before the first data collection commencement, a pretest was conducted with 29 subjects from various target facilities outside the main study. The pretest was conducted to familiarize enumerators with the interview process’s administration and ensure consistency. Debriefing sessions were held with the pretest field staff, and the questionnaire was revised based on the pretest findings.

The selected hospitality workplaces’ owners/managers were approached and informed about the study. Their permission/collaboration was sought to collect data from their sites. Once permission was granted, the interviewers contacted the participants and assured them of their eligibility for participation. Then, the survey was conducted using a prepared questionnaire in English and translated into Amharic (the local language). The questionnaire was adopted from other literature and included a range of questions on sociodemographic characteristics, awareness, attitude, sexual violence experiences, and associated factors of sexual violence in the workplace. It also had questions about sexual violence experiences related to verbal, non-verbal, and physical violence. From the evening until 8:00 p.m., quantitative and qualitative data were collected. The respondents were interviewed while they were off duty. Daily, completed questionnaires were cross-checked for consistency and completeness. Two public health professionals and the principal investigator closely supervised the data collection process.

Data were collected using different methods and sources for the qualitative part, including in-depth interviews and focus group discussions. Multiple data collection methods were used to reduce biases introduced using a single approach [30, 31]. It also enables us to avoid the limitations of using any single way. FGDs were first directed to place issues in a group setting where comfortable adult females could share their experiences and ideas. Following the FGDs, the IDIs were conducted with separate female workers, and they were able to speak out more in-depth about their workplace experiences. In-depth interviews and focus group discussions were conducted to understand the individual and group perspectives on WSV perceptions and experiences at work.

Data were collected from January to August 2019, as indicated in our prior investigations [17, 18]. The data collection time was extended due to the difficulty in reaching women due to their extremely long working hours. The purpose of the interview guidelines was to elicit debate among study participants. The themes covered in the IDIs and FGDs were identical, and the issues were women’s perceptions of WSV. Five people with similar demographic profiles were used to pretest all the guides. The pretest was designed to ensure that the rules and interview methodologies were suitable for local settings. These people were not included in the research. All of the discussions were held in a single room.

The FGDs and IDIs were conducted by four researchers (two for each), with one facilitating the conversations. Simultaneously, the other assisted in gathering the women and taking notes as needed. Each participant was assigned over the phone, as in our prior investigations [17, 18]. All interviews were audio-recorded with the participant’s permission. Each interview and focus group lasted 60 to 105 min on average, with an average of 80 min. FGDs participants were served tea, coffee, water, and soft drinks to thank them for their time and cover their transportation costs.

### Data management and analysis

After completing the data entry into Epidata version 7, the data were exported to the SPSS version 24 software package and Excel for data checking and cleaning. Then, it was exported to STATA 15 for analysis. Cleaning was accomplished through the calculation of frequencies and sorting. Binary logistic regression was used to perform a bi-variate analysis between dependent and independent variables. A *P* value < 0.25 was used as a criterion to select candidate variables for multi-variate analysis. Multi-variable logistic regression analysis was done to adjust for possible confounding variables. The significance of the associations was judged using a *P* value < 0.05 with a 95% confidence interval (CI) for OR (odds ratio). The findings were presented in text, tables, and graphs.

All recorded interviews and FGDs were transcribed into a composed text for the qualitative part. The texts were cross-checked with audio files for accuracy and consistency before coding. The copies were prepared by a research assistant, a university graduate with experience conducting qualitative research and preparing for information aggregation. The first author (MD) reads a sub-sample of transcripts to check the consistency of the transcripts. Data were analyzed following a thematic analysis approach [32, 33]. We study and re-read the descriptive information to become acquainted with the data to obtain codes for thematic analysis. The analysis method combined prior codes with data-driven codes based on the research question. An open coding method was used to perform information-driven codes, categorizing small codes. The small codes were grouped to form critical themes, with emerging issues serving as analysis categories [34]. To ensure the reliability of the coding, the principal investigator and the research assistant coded a sample of transcripts from each interview category independently. They held discussions to reach an agreement on a final code list. The codes were added to subsequent transcripts using Atlas-ti version 8.4. There were vital themes such as pressure, retaliation, sexually sensitive movies, pictures, and forced sex. These key themes served as the foundation for the thematic framework.

## Results

### Sociodemographic characteristics

Out of the expected 406 respondents, 401 agreed to participate, yielding a response rate of 98.8%. The respondents’ ages ranged from 18 to 35, with a mean and standard deviation (SD) of 22.07 (± 2.83). All respondents live within a 5 km radius of their workplace and walk less than 60 min by foot (Table 1). Similarly, 45 female employees participated in the 6 FGDs and 10 IDIs. The average length of record for each IDI and FGD was 80 min. The women’s ages ranged from 18 to 37 years.

**Table 1:** Sociodemographic and socioeconomic characteristics of the study participants working in Bahir Dar City Administration hospitality workplaces, January to August 2019 (n = 401)

Variable	Frequency (no)	Percent (%)
Age		
18–25 years	357	89
26–30 years	39	9.7
31–35 years	5	1.2
Religion		
Orthodox	387	96.5
Muslim	3	0.7
Protestant	11	2.7
Educational level		
Able to read and write	35	8.7
Primary education	116	28.9
Secondary education	176	43.9
College and above	74	18.5
Ethnicity		
Amhara	378	94.3
Agew	17	4.2
Others (Oromo, Tigre)	6	1.4
Employment status		
Permanent	129	32.2
Contract	255	63.6
Part-timer	17	4.2
Work experience		
≤ One year	231	57.6
2–4 years	147	36.7
≥ Five years	23	5.7
Living accompany		
Alone renting a house	200	49.9
At working place with coworkers	34	8.5
With family	54	13.5
With friends renting a house	49	12.2
With boyfriend/husband	64	15.9
The place they grew		
Bahir Dar City	61	15.2
Another City	160	39.9
Rural	180	44.9
Working area		
Reception	23	5.7
Waitressing	378	94.3

#### Awareness of sexual violence

Among the total respondents, 284 (70.8%) disclosed that they had heard about sexual violence. Among those who had awareness, 206 (72.5%) got the information from their friends, and 108 (26.9%) heard it from their village. Only 88 (21.9%) disclosed that they had heard about workplace sexual violence among the respondents. However, only 24 (6%) got information from their employers. Similarly, of all respondents, only 15 (3.7%) got training about WSV in their workplace. Additionally, 322 (80.3%) answered that they would report it to the police (Table 2).

**Table 2:** Awareness of workplace sexual violence among women working in Bahir Dar city hospitality workplaces, January to August 2019

Variable	Frequency	%
Have you heard about sexual violence in the last year?		
No	117	29.17
Yes	284	70.83
From whom did you get information about Sexual Violence?		
Friends	206	72.5
Media	53	18.7
Family	5	1.8
Others	20	7
Have you heard about sexual violence in your village in the last year?		
No	293	73.1
Yes	108	26.9
Have you heard about workplace sexual violence in the last year?		
No	313	78.1
Yes	88	21.9
Have you been informed about workplace violence by your employers?		
No	377	94
Yes	24	6
Have you been trained about workplace sexual violence in your workplace?		
No	386	96.3
Yes	15	3.7
Have you heard about the possibility of reporting WSV experiences to the police?		
No	79	19.7
Yes	322	80.3

#### Perception of respondents

Only 97 (24.4%) of all study participants strongly agreed that they would report the incident to the police, and only 43 (10.7%) believed that exposing the WSV act would affect work efficiency (Table 3).

**Table 3:** Perception of workplace sexual violence among women working in Bahir Dar City hospitality workplaces, January to August 2019

Perception	Strongly agree	Agree	Neutral	Disagree	Strongly disagree
	No (%)	No (%)	No (%)	No (%)	No (%)
If I face sexual violence in my workplace, I would report it to the police	97 (24.2)	277 (69.1)	10 (2.5)	16 (4)	1 (0.2)
Exposing workplace sexual violence will influence work efficiency	43 (10.7)	213 (53.1)	12 (3)	121 (30.2)	12 (3)
Exposing workplace sexual violence creates a non-conducive work environment	51 (12.7)	209 (52.1)	13 (3.2)	119 (29.7)	9 (2.2)
Workplace sexual violence is one of the opportunities to get a boyfriend	16 (4)	108 (26.9)	53 (13.2)	173 (43.1)	51 (12.7)
Workplace sexual violence is an everyday social activity; it is not bad	9 (2.2)	126 (31.4)	31 (7.7)	136 (33.9)	99 (24.7)
Sexual violence in hospitality workplaces is expected and acceptable	16 (4)	144 (35.9)	26 (6.5)	127 (31.7)	88 (21.9)
Since our income is a customer, we should tolerate sexual violence from them	97 (24.2)	170 (42.4)	58 (14.5)	39 (9.7)	37 (9.2)
Hospitality workplaces should have clear policies and regulations about WSV	164 (40.9)	255 (56.1)	4 (1)	3 (0.7)	5 (1.2)

The participants in the qualitative study reported that sexual violence was a common issue in their workplaces. Though they did not classify the events into distinct categories, they perceived different sexual violence incidents in their workplaces. For example, most participants mentioned that exaggerated tips and inappropriate promises of benefits in exchange for sexual favors were used to get them to participate in unwanted sexual behaviors at work.

Sexual violence is a situation in which women working in the hospitality industry are forced to engage in sexual activity against their choice. They are most likely to be duped by tips, another unneeded present, or the inappropriate promise of benefits in exchange for sexual favors. (25 years, IDI, four years in a cafeteria)

The study participants also considered the activities carried out by the supervisors or the owner as one part of sexual violence. These activities included promoting and proposing new jobs and donating money in exchange for sexual favors.

Sexual violence is an action by a supervisor or owner that can be explained by offering money, promising awards, and promoting a better employment position with a higher income scale in exchange for advanced sexual favors. (FGD five)

Participants also perceived sexual acts of violence as perpetrator activities such as threatening women’s relatives, firing from jobs, complaining, or falsely accusing about the service provided to the immediate in exchange for sexual favors.

Sexual violence exposes my vulnerable side, making it difficult to resist sexual advances. My soft parts are my financial difficulties and having a family member or a loved one to take care of. Thus, I believe sexual violence is represented by threatening to harm a loved one, reporting to my direct supervisors about my service provider performance, threatening me to fire from a job, and refusing me to pay for services unless we fulfill sex requests. (22 years, IDI, four years in a cafeteria)

#### Prevalence of workplace sexual violence

Three hundred eighty-seven (96.5%) of all the participants anticipated sexual violence in hospitality workplaces. Furthermore, 184 (45.9% (95% CI 41, 50.8)) of all respondents had been sexually violated.

In terms of the types of WSV, 178 (96.8%) had their sexual rights violated verbally, 172 (93.5%) had their sexual rights violated physically, and 184 (45.9%) had their sexual rights harassed. Furthermore, 18 (9.58%) were raped, 91 (49.5%) were attempted to be raped, 167 (90.5%) were a quid pro quo, and 149 (80.8%) were unwanted physical contact, and 40 (21.7%) were forced to kiss.

#### Perpetrators of sexual violence

Customers were responsible for 155 (84.2%) of all WSV victims, followed by managers (4.2%), owners (6.3%), supervisors (3.6%), coworkers (10.4%), and others (6.3%). In terms of age, 96 (52.2%) of the perpetrators were between the ages of 26 and 35; 61 (33.2%) were between the ages of 37 and 45; 19 (10.3%) were between the ages of 18 and 25, and 8 (4.3%) were over 45.

#### Experience with sexual violence

**Fig. 2:**
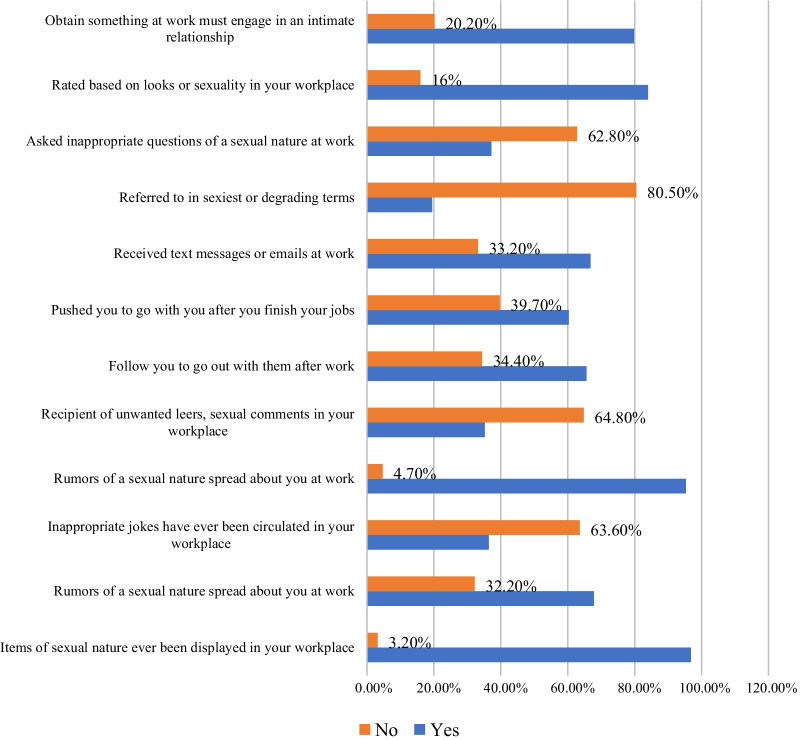
Experience of workplace sexual violence in Bahir Dar City Administration hospitality workplaces, January to August 2019

Among all participants, 96.8% witnessed displayed items of a sexual nature in their workplaces, and 95.3% experienced the spread of rumors of a sexual nature in their workplaces (Fig. 2).

Participants in the qualitative study mentioned that perpetrators touched women’s sexually sensitive parts, made random sexual jokes, verbal, sexual requests, repeated requests for sexual mating, sexual solicitation, sexual intimidation, sexual prodding, and requested a phone call.

In my workplace, I faced activities such as hugging, touching breasts and touching hips while I was at work. (20 years, IDI, two years experience in a restaurant)

Another participant added:

Touching my breasts, hips, and genitalia, slapping my hips and face, requesting sexual intercourse, commenting on my physical characteristics, inviting me for dinner, and requesting me for sex were all examples of activities that I faced in my workplaces. (21 years old, IDI, one year in a cafeteria)

Participants also mentioned that they were invited to watch pornographic movies/pictures, got messages of sexual requests to pay bills, mistreated, disparaged, tried to be abducted and raped, slapped, kicked, pinched, and insulted verbally.

We believed that sexual violence is explained by […], winking, and undermining us considering our gender. (FGD one)

Other participants added:

O! We think sexual violence is rape or abduction. (FGD four)

As we faced so far, sexual violence was spitting drinks, slapping, pinching, caressing, talking unnecessary sexual talks, and talking and distributing false things about us to the manager. (FGD two)

#### Behaviors, family environment, and work environment

Among all respondents, 123 (43.1%) stated that the rights and responsibilities of employees are known in their workplace, 48 (12%) reported problems as a result of refusing workplace sexual violence, and 155 (38.7%) believed that the afternoon shift is more dangerous for sexual violence (Table 4).

**Table 4:** Behaviour, work-related factors, and family environment among waitresses working in *a hospitality workplace in Bahir Dar City from* January to August 2019

Experience	No No (%)	Yes No (%)
Have you ever been drinking alcohol in the last six months?*	312 (77.8)	89 (22.2)
Have you ever been using drugs in the last six months?	399 (99.5)	2 (0.5)
Did you practice sexual fantasies supportive of SV in the last 6 months?*	377 (94)	24 (6)
Did you have impulsive and anti-social tendencies?*	392 (97.8)	9 (2.2)
Did you face sexual abuse as a child?*	375 (93.5)	26 (6.5)
Did you witness family violence as a child?*	286 (71.3)	115 (28.7)
Did you have sexually aggressive and delinquent peers?*	328 (81.8)	73 (18.2)
Did you have a family environment characterized by poverty & few resources?*	200 (49.9)	201 (50.1)
Did you have a particularly robust relationship or family environment?*	26 (6.5)	375 (93.5)
Did you have an emotionally unsupportive family?*	112 (27.9)	289 (72.1)
Did your family consider their honour more important than your health & safety?*	307 (76.6)	94 (23.4)
Did you face poverty mediated through forms of crises of male identity?	341 (85)	60 (15)
Did you face a lack of employment opportunities?	204 (50.9)	197 (49.1)
Did you face a lack of support from the police or other judicial body?	333 (83)	68 (17)
Did you tolerate sexual assault within your community?	258 (64.3)	143 (35.7)
Did you have weak community sanctions against perpetrators of SV?	271 (67.6)	130 (32.4)
Did you have societal norms supportive of sexual violence?	309 (77.1)	92 (22.9)
Did you have societal norms supportive of male superiority?	214 (53.4)	187 (46.6)
Did society have weak laws and policies related to SV?	292 (72.8)	109 (27.2)
Did your community have weak laws and policies related to gender equality?	298 (74.3)	103 (25.7)
Did you witness a high level of crimes and other forms of violence in your community?	249 (62.1)	152 (37.9)
Did you wear uniforms in your workplace?	68 (17)	333 (83)
What type of uniform did you wear?		
Mini		250 (74.9)
Skirt		10 (3)
Trouser		30 (9)
Others		43 (13.1)
Do you like the uniform?	127 (38)	206 (62)
Did you work a shift?	87 (21.7)	314 (78.3)
Which shift are you working on?		
Morning		199 (63.2)
Afternoon		61 (19.4)
Evening		9 (2.9)
Others		45 (14.6)
Which shift do you think exposure to sexual violence?		
Morning		5 (1.2)
Afternoon		155 (38.7)
Evening		123 (30.7)
Others		34 (8.5)
Do not know		84 (20.9)
Did you face problems due to the refusal of sexual violence?	353 (88)	48 (12)
Are the rights and responsibilities of employees known in your workplace?	278 (56.9)	123 (43.1)

* Casey and Masters [22]

### Factors associated with workplace sexual violence

The bi-variate analysis displayed statistically significant associations between workplace sexual violence and age; monthly income; awareness of sexual violence and workplace sexual violence; the habit of alcohol drinking; a history of family violence as a child; having sexually aggressive and delinquent peers; and growing up in a low-income family environment. Additionally, an emotionally supportive work environment, family consideration of honor over child safety and health, lack of employment opportunity, communities’ weak sanctions against sexual violence, societal norms supportive of sexual violence, a societal norm of supporting male superiority, weak laws, and policies of society related to sexual violence, witnessing high crimes and other forms of violence in the community, and educational status were significantly associated with WSV. Furthermore, in the multi-variable logistic regression analysis, awareness of sexual violence and workplace sexual violence, witnessing family violence as a child, family consideration of honor over child safety and health, lack of employment opportunities, education, and an emotionally supportive work environment were all significant.

Waitresses aware of sexual violence were nearly two times more likely to experience WSV than their counterparts (AOR = 1.89, 95% CI 1.09, 3.27). Additionally, waitresses aware of workplace sexual violence were 2.02 times more likely to experience WSV than their counterparts (AOR = 2.02, 95% CI 1.12, 3.66). Waitresses who witnessed family violence as children were also 1.75 times more likely to experience sexual violence than their counterparts (AOR = 1.75, 95% CI 1.02, 2.99). Moreover, waitresses raised in families who considered their honor more important than their children’s safety and health were 2.07 times (AOR = 2.07, 95% CI 1.18, 3.64) more likely to experience WSV than their counterparts. Furthermore, waitresses who lacked employment opportunities were 1.83 times more likely to be exposed to WSV (AOR = 1.83; 95% CI 1.14, 2.94) than their counterparts.

However, the waitresses who had emotionally supportive work environments were 55% less likely to experience WSV than their counterparts (AOR = 0.45, 95% CI 0.27, 0.77). Similarly, those who had primary education, secondary education, and were educated more than college levels were 82% (AOR = 0.18, 95% CI 0.07, 0.48), 71% (AOR = 0.29, 95% CI 0.15, 0.59), and 60% (AOR = 0.40, 95% CI 0.21, 0.57), less likely to experience WSV, respectively, than those who could only read and write (Table 5).

**Table 5:** Bivariate and multi-variate logistic regression analysis output of factors associated with sexual violence among women hospitality workplace employees, Bahir Dar, January to August 2019

Variables	WSV		COR (95%CI)	*P* value	AOR (95%CI)	*P* value
	No	Yes				
Age	187	30	1		1	
	144	40	1.088 (1.014,1.169)	0.019	1.03 (0.94, 1.13)	0.550
Monthly income	165	52	1		1	
	126	58	1.001 (1.000,1.001)	0.004	1 (1.000, 1.001)	0.193
Have you heard about sexual violence in the last year?						
No	84	133	1	< 0.001	**1 **	
Yes	33	151	2.89 (1.82, 4.60)		**1.89 (1.09, 3.27)**	**0.023**
Have you heard about workplace sexual violence in the last year?						
No	189	28	1		**1**	
Yes	124	60	3.27 (1.98, 5.40)	< 0.001	**2.02 (1.12, 3.66)**	**0.020**
Have you ever been drinking alcohol in the last six months?						
No	185	32	1		1	
Yes	127	57	2.60 (1.60, 4.30)	< 0.001	1.51 (0.84, 2.71)	0.17
Did you witness family violence as a child?						
No	180	37	1		**1 **	
Yes	106	78	3.58 (2.26, 5.67)	< 0.001	**1.75 (1.02, 2.99)**	**0.041**
Did you have sexually aggressive and delinquent peers?						
No	194	23	1		1	
Yes	134	50	3.15 (1.83, 5.40)	< 0.001	1.86 (0.99, 3.50)	0.056
Do you have a family environment characterized by poverty and few resources?						
No	127	90	1			
Yes	73	111	2.15 (1.44, 3.20)	< 0.001	1.38 (0.83,2.30)	0.215
Do you have an emotionally supportive work environment?						
No	178	39	1		**1 **	
Yes	111	73	0.333 (0.211, 0.525)	< 0.001	**0.45 (0.27, 0.77)**	**0.003**
Does your family consider their honour more important than your health and safety?						
No	182	35	1		**1 **	
Yes	125	59	2.45 (1.53, 3.95)	< 0.001	**2.07 (1.18, 3.64)**	**0.011**
Did you face a lack of employment opportunities?						
No	137	80	1		**1 **	
Yes	67	117	2.99 (1.99, 4.50)	< 0.001	**1.83 (1.14, 2.94)**	**0.012**
Does your community have weak sanctions against sexual violence?						
No	164	53	1			
Yes	107	77	2.23 (1.45, 3.41)	< 0.001	0.90 (0.50,1.64)	0.742
Do you have societal norms supportive of sexual violence?						
No	184	33	1		1	
Yes	125	59	2.63 (1.62, 4.67)	< 0.001	1.39 (0.73, 2.67)	0.317
Do you have societal norms supportive of male superiority?						
No	141	76	1		1	
Yes	73	111	2.82 (1.88, 4.24)	< 0.001	1.55 (0.95,2.51)	0.078
Does your society have weak laws and policies related to Sexual Violence?						
No	167	50	1		1	
Yes	125	59	1.58 (1.01, 2.50)	< 0.044	0.97 (0.52,1.83)	0.927
Did you witness a high level of crimes and other forms of violence in your community?						
No	152	65	1		1	
Yes	97	87	2.1 (1.39, 3.16)	< 0.001	1.14 (0.68,1.90)	0.613
Educational status						
Able to read and write	23	12	1		1	
Primary education	79	37	0.90 (0.40, 2.00)	0.79	**0.18 (0.07, 0.48)**	**0.001**
Secondary education	93	83	1.71 (0.80, 3.65)	0.17	**0.29 (0.15, 0.59)**	**0.001**
College and above	22	52	5.53 (1.92, 10.68)	0.001	**0.40 (0.21, 0.77)**	**0.005**

Bold indicates the variables that are significant during AOR analysis

## Discussion

According to the findings of this study, 45.9% (95% CI: 41%, 51%) of the waitresses had been subjected to workplace sexual violence, with verbal sexual violence and attempted rape being the most common forms. The prevalence of WSV found in our study is consistent with what has been found in Ghana [35] and Uganda [36]. However, it is lower than the figure reported by Topping [37]. The difference observed could be attributed to the variation in the population of interest in our study, indicating that WSV is a major human resources issue that is more common in host workplaces. It also implies that further studies in similar contexts are needed to strengthen outcomes, which could be achieved by conducting similar studies in different parts of the country.

Identifying the risk factors for WSV may help target interventions to reduce sexual violence in specific occupations. Thus, the second goal of this study identified exposing factors such as awareness of sexual violence and workplace sexual violence, witnessing family violence as a child, lack of employment opportunities, and family consideration of their honor rather than their child’s health. On the other hand, the identified prevention factors were an emotionally favorable environment and formal education enrolment.

The study found that those who were aware of sexual violence and workplace sexual violence were more likely to experience WSV than those who were not. This finding is consistent with findings from Nigeria [38, 39] and the US [40]. This finding suggests that knowing the socially constructed meaning of sexual violence may improve waitress judgment based on their experience, perpetrator characteristics, and the situations. As a result, waitresses assess the situation while being familiar with the components according to the interpreted definition. Thus, WSV among waitresses who were aware may not be necessarily higher than their counterparts but may be due to a difference in their capacity to evaluate what constitutes sexual violence. Therefore, when studying the relationship between WSV and participant awareness, socio-cultural factors and waitress personalities need to be considered.

The qualitative study revealed that the results were consistent with the previous study [41]. However, most respondents did not know about sexual violence at work. Likewise, consistent with the Zimbabwean research [42], waitresses in this study had inadequate awareness of workplace sexual violence before or during the hiring process. Waitresses must be aware of and skilled in dealing with SV outside of the hospitality workplace. Aside from a lack of exposure to sexual and reproductive health-related training, including WSV, Ethiopian waitresses frequently fail to differentiate between forms of WSV [17]. Thus, for waitresses to be aware of the WSV, unique approaches and system reforms must be implemented to enhance their knowledge and ability to work. These approaches must include training for waitresses to boost their confidence in preventing WSV.

Waitresses who did not have job opportunities were almost twice as likely to be exposed to sexual violence at work. This finding could be explained by the fact that unemployed waitresses may be willing to work in low-wage jobs, which are more likely to occur in smaller, less formalized workplaces with no official complaint mechanisms [43]. This situation also forces waitresses who have no other employment options to work for tips in isolated settings, in workplaces that lack formal mitigation techniques, and in workplaces that are male-dominated and have significant power differentials. These workplace characteristics may, in turn, expose waitresses to WSV. Consequently, this finding points to the need to improve occupational safety and health in spite of new employment opportunities, especially for women.

Waitresses who witnessed family violence as children were also nearly two times more likely to experience sexual violence than those who did not notice it. This finding is consistent with findings of high school students in southern Ethiopia [44], Meda Walabu University [45], and Butajira Town [46], and a meta-analysis that was conducted to investigate the association between child witnesses to domestic violence and child problems in later life [47]. “Family violence or domestic violence is a pattern of abusive behaviors in a relationship used to gain and maintain control and power over another person” [48]. Examples of abusive behaviors include physical abuse, sexual abuse, emotional abuse, psychological abuse, and technological or financial abuse [48]. Domestic violence has a devastating effect on children who witness it, and these children are more likely to be mistreated themselves [49]. Evidence indicates that children who witnessed family violence have much lower psycho-social outcomes than those who did not witness family violence [50]. Therefore, there may be a psychological connection between sexual violence in the workplace and witnessing family violence in childhood. This suggests that a qualitative design should be used to investigate the specific relationship.

Furthermore, this study found that waitresses raised in a family that valued their honor over their children’s safety and health had more than twice the odds of experiencing WSV as waitresses raised in a family that valued child safety and health over their honor. Family honor refers to the perceived quality of worthiness and respectability, which influences their social standing and self-evaluation both corporately and individually [51, 52]. In patriarchal societies like Ethiopia, the family is regarded as the primary source of honor, and the community places a high value on honor and family relationships [53]. Family members’ behavior reflects family honor and how the family perceives itself and others [53]. Family honor can be affected by various factors and areas, including social status, religion, clothing, food, education, employment or career, property such as real estate, and marriage. Therefore, societies where “family honor” is highly valued generally impose a high degree of restriction on the freedom of their daughters to work as waitresses [54, 55]. If a family believes they have been abused or treated disrespectfully because of their daughter, they have the right to defend their honor or seek reparation or vengeance [56]. As a result, the daughters will tolerate and remain silent about exposing the WSV they face, escalating further sexual violence and sequela. This finding suggests that the family-to-child relationship could be used as an intervention to prevent WSV.

Waitresses who worked in emotionally supportive work environments, on the other hand, were 55% less likely to experience WSV than their counterparts. This finding is consistent with findings from China [57, 58] and Denmark [59]. This finding could be explained by the fact that organizational-level psycho-social climate is positively associated with better workers’ health, both physical and psychological, as well as the better performance at work [60–64], and is also regarded as a predictor of a better psycho-social work environment [2, 60, 64–67]. Hence, lower WSV and, as a result, better workers’ mental health may be associated with a favorable psycho-social climate [68]. Furthermore, employees in high psycho-social climate workplaces were more willing to respond to and resolve WSV [2, 69], which moderates the relationship between WSV and mental health problems and job disengagement [2, 68]. Thus, psycho-social support is essential for employees who are victims of WSV.

Waitresses with primary and secondary education and more than a college education were 82%, 71%, and 60% less likely to experience workplace sexual violence than those who could only read and write. This finding is consistent with the study findings in Mekelle [70]. This is perhaps because less-educated waitresses are less likely to earn a competitive salary. In a competitive workplace such as the hotel industry, waitresses with more education and work experience will be hired first. In the worst-case scenario, waitresses with lower levels of education face lower-paying or unemployed jobs. This can create dependency for those who are less educated if they tend to earn less money and are unable to support themselves. If sexual violence already exists in the workplace, the perpetrator has a benefit in exploiting the victim’s vulnerability as a result of lack of education and limited income.

Furthermore, education makes it possible for people to learn new things. Greater educational attainment offers benefits other than knowledge. The expertise demanded by various professions goes hand in hand with a better understanding of specific subjects. Thus, an individual’s social standing is determined by multiple factors, including professional status, income level, and educational level. Individuals can also meet new people and gain new perspectives on life through their college experience. This includes developing fresh air on acceptable and unacceptable relationships. It is also believed that education increases an individual’s ability to rationalize in everyday life, allowing them to make better life decisions. Those with more education are also thought to be better communicators, which may serve as a protective factor against workplace sexual violence [71].

In summary, the findings of this study and our systematic review [27] revealed that workplace sexual harassment (WSH) is the most common type of WSV. These findings imply that efforts to address workplace sexual harassment in hospitality settings must be based on a thorough understanding of the numerous intersecting inequalities. Thus, future studies should use a valid and reliable measurement tool to understand and measure WSH. The magnitude, the individual and organizational factors, and the outcomes and relationships of each effect should all be determined.

Despite gaining essential insights on WSV among waitresses, this study has inherent limitations. The first is that the study design nature, i.e., cross-sectional study, will not tell us the time between the factors and the outcome variables. The second limitation is that the problem’s burden may be underestimated since there are dropouts and absentee victims.

## Conclusions

Workplace sexual violence was prevalent among waitresses in the study area. Workplace sexual violence was significantly associated with awareness of sexual violence and workplace sexual violence; witnessing family violence as a child; working in an emotionally supportive work environment; family consideration of honor over their child’s health; lack of employment opportunity; and educational status. More large-scale studies involving male hospitality workplace workers are needed to understand better the factors contributing to WSV in Ethiopia. These require the attention of governmental, non-governmental, and civic organizations and other responsible bodies to tackle the study area’s factors aspects.

Furthermore, the themes identified here represent waitresses’ perceptions, prevalence estimates, and analysis of risks related demographic characteristics. It is beneficial to provide waitresses with practical support and security and to promote gender-equitable attitudes among waitresses, customers, and coworkers. Likewise, organizational anti-sexual violence policies and strategies must be developed and implemented.

## Supplementary Information

**Additional file 1.** Focus Group Guide for women hospitality workplace workers.

**Additional file 2.** In-depth interview guide for women hospitality workplace workers.

## Data Availability

All data generated or analyzed during this study are included in this manuscript [and its addtional files].

## Abbreviations

AIDS: Acquired Immune Deficiency Syndrome
BCC: Behavioral Change Communication
CIRHT: Center for International Reproductive Health Training
CSW: Commercial Sex Worker
FGDs: Focus Group Discussions
IRB: Institutional Review Board
KIIs: Key informant Interviews
LMICs: Low-income and Middle-income Countries
MoSHE: Ministry of Science and Higher Education
Ph.D.: Doctor of Philosophy
SH: Sexual Harassment
WSH: Workplace Sexual Harassment
WSV: Workplace sexual violence
