# Supplementary material for: Factors associated with sexual violence among waitresses working in Bahir Dar City, Ethiopia: a mixed-method study
Source: BMC Womens Health. 2022 Jun 6;22:209. doi: 10.1186/s12905-022-01806-x (PMC9172177; doi:10.1186/s12905-022-01806-x)
Supplement: Supplementary file 1 — Additional file 1. Focus Group Guide for women hospitality workplace workers. [file 12905_2022_1806_MOESM1_ESM.docx]

**Part I: General Information**

1. Date of the interview**:**/____/______/________/
2. Audio file no: _______________________ code no___________________
3. Venue: Blue Nile Hotel­­
4. Number of the participants: ___________________________
5. Category of the interview: Women working in hospitality workplaces

**Part II. Respondents Background**

(Do not write their name or code as P1, p2, p3…. p12 under the column headed P. code)

| NO | P. code | Age | Educational status | Roles | Birth place | Kebele |
| --- | --- | --- | --- | --- | --- | --- |
| 1. |  |  |  |  |  |  |
| 2. |  |  |  |  |  |  |
| 3. |  |  |  |  |  |  |
| 4. |  |  |  |  |  |  |
| 5. |  |  |  |  |  |  |
| 6. |  |  |  |  |  |  |
| 7. |  |  |  |  |  |  |
| 8. |  |  |  |  |  |  |

| No | Questions | Remark |
| --- | --- | --- |
| 101 | How do you understand sexual harassment? |  |
| 102 | Can you tell us about any related acts of sexual violence to the women working in Hospitality workplaces? Probes:  -Sexual jokes, Attempted to draw you into sexual banter, Made offensive sexual remarks in public or private, Attempted to make a romantic relationship with you, Treated you differently because of your sex, Made you feel like you were being bribed with rewards or consequences, Touched in an uncomfortable way |  |
| 103 | How frequently do acts of sexual harassment against women working in hospitality workplaces occur in your workplace? |  |
| 104 | Who are the potential perpetrators of sexual harassment the women working in hospitality workplaces?  Probe: customer, supervisor manager, co-worker |  |
| 105 | Where do you go when you seek help concerning sexual harassment perpetration? |  |
| 106 | What do you think is the cause of sexual harassment against women working in hospitality workplaces? **Probe:** Behavior, Clothing, |  |
| 107 | What do you think is/are the impact of sexual harassment against women working in hospitality workplaces? Probe: Sexually transmitted disease, Mental health, Suicidal behaviour, Social ostracisation. |  |
| 108 | Anything else you would like to add? |  |
