# Supplementary material for: Factors associated with sexual violence among waitresses working in Bahir Dar City, Ethiopia: a mixed-method study
Source: BMC Womens Health. 2022 Jun 6;22:209. doi: 10.1186/s12905-022-01806-x (PMC9172177; doi:10.1186/s12905-022-01806-x)
Supplement: Supplementary file 2 — Additional file 2. In-depth interview guide for women hospitality workplace workers. [file 12905_2022_1806_MOESM2_ESM.docx]

**Guiding Questions for women working in hospitality workplaces for the In-depth Interview**

**Part I: General information**

101: Date of the interview**: /**____/______/________/

102: Code no/___________________/

103: Kebele: /__________________________________/

104: Category of the interview**: -**women working in hospitality workplaces

| **Part II: Socio-Demographic Characteristics Respondents** | | | |
| --- | --- | --- | --- |
| NO | **Questions** | **Remark** | |
| 201 | Age: /_______________/ complete year |  | |
| 202 | Profession:/ ______________________________________________________/ |  | |
| 203 | Educational status: /________________________________________________/ |  | |
| 204 | Position held: /waiter/ |  | |
| 205 | Service year:/__________________________________________/ |  | |
| 206 | **Opening questions:**   1. What is your work experience as a woman working in hospitality workplaces? |  | |
| **Part III: Sexual Harassment Related Questions** | | | |
| 207 | How do you understand sexual harassment?  How do you understand the characteristics of sexual harassment? | |  |
| 208 | Tell me about any incidents where you were made non-comfortable or treated non-appropriately.  Probe: What exactly happened? What correctly did he do/say? Verbal? How explicit? Physical? In what context did it occur? Over what period did it occur? Frequency? Was anyone else aware of the behaviour? Any other incidents? | |  |
| 209 | - Who harassed you? What was your relationship with the sexual harasser before and during the sexual harassment? How much is the influence of the harasser on this facility? Did the harasser have the power to take the business elsewhere? - How much of your personal life is reliant on the harasser? If you lost your job, would you suffer? | |  |
| 210 | - What is your opinion on sexual harassment in the hospitality industry? - Do you consider it to be a problem within the industry? - Have you ever been touched by the issue? How? | |  |
| 211 | Is there anything else you would like to add? | |  |
